# Ultrasound-Guided Peripheral Nerve Block for Emergency Below-Knee Amputation in a High-Risk Patient With Coagulopathy: A Case Report

**DOI:** 10.7759/cureus.87989

**Published:** 2025-07-15

**Authors:** Shashank Krishnakumar, Vinod Krishnagopal, Raj Murugan, Roshni Iyer

**Affiliations:** 1 Anaesthesiology, Apollo Hospitals, Chennai, IND; 2 Anaesthesiology, Sree Balaji Medical College and Hospital, Chennai, IND; 3 Anaesthesiology, Parvathy Hospitals, Chennai, IND

**Keywords:** anticoagulation, below-knee amputation, diabetic foot, ultrasound-guided peripheral nerve block, ultrasound-guided regional anesthesia

## Abstract

Emergency below-knee amputation (BKA) in patients with significant comorbidities and coagulopathy presents formidable anesthetic challenges, as a central neuraxial blockade and general anesthesia are frequently contraindicated. We report the case of a 57-year-old female patient with uncontrolled diabetes mellitus, chronic kidney disease, coronary artery disease, and an elevated international normalized ratio (INR) of 1.6, necessitating urgent BKA for a septic diabetic foot ulcer. Ultrasound-guided peripheral nerve blocks (PNBs, popliteal sciatic and femoral) have been successfully employed to achieve effective intraoperative anesthesia and postoperative analgesia without bleeding complications or hemodynamic instability. This case underscores the safety and efficacy of ultrasound-guided PNBs as the preferred anesthetic strategy in high-risk patients with coagulopathy, offering a safer alternative to conventional methods and minimizing perioperative morbidity.

## Introduction

Below-knee amputation (BKA) is a critical surgical intervention for limb-threatening conditions such as diabetic foot infections complicated by sepsis or osteomyelitis [1]. In emergency settings, patients with multiple comorbidities, including cardiovascular disease, renal impairment, and coagulopathy, pose significant anesthetic challenges. Central neuraxial blockade is contraindicated in patients with elevated international normalized ratio (INR) due to the risk of spinal hematoma, whereas general anesthesia may exacerbate hemodynamic instability in patients with compromised cardiac function [2,3]. Ultrasound-guided peripheral nerve blocks (PNBs) have transformed regional anesthesia by enabling precise visualization of neural and vascular structures and reducing complications even in anticoagulated patients [4,5]. This case report details the successful application of ultrasound-guided PNBs in a high-risk patient undergoing emergency BKA, highlighting their role as safe and effective anesthetic modalities in complex clinical scenarios.

## Case presentation

A 57-year-old female patient, weighing 71 kg, presented with a two-month history of a non-healing diabetic foot ulcer on the right foot, complicated by severe neuropathic pain, cellulitis, osteomyelitis, and systemic sepsis, necessitating urgent BKA. She reported an inability to ambulate for one month. Her medical history included poorly controlled diabetes mellitus (30 years), chronic kidney disease (one year), coronary artery disease with heart failure (15 years), spinal tuberculosis, and systemic hypertension (one year). Medications included insulin (human mixtard 8 units subcutaneously thrice daily), aspirin 75 mg daily, clopidogrel 75 mg daily, ramipril 2.5 mg daily, and sodium bicarbonate 500 mg daily. 

On examination, the patient was conscious, oriented, febrile (temperature 37.8°C), tachypneic (respiratory rate 24 breaths/minute), and tachycardic (heart rate 112 beats/minute). She maintained 97% oxygen saturation at 5 L/min via a non-rebreathing mask but was hypotensive (blood pressure 90/60 mmHg, measured in the left arm, supine). Physical findings included bilateral pitting pedal edema, pallor, and coarse crepitations on chest auscultation. The right lower limb exhibited edema, blackish discoloration, and a nonhealing ulcer over the medial malleolus with absent peripheral pulses.

Laboratory investigations revealed hemoglobin of 9 g/dL, an INR of 1.6, and a normal platelet count (2.73 × 10^9/L). The bleeding time (two minutes 35 seconds) and clotting time (four minutes 45 seconds) were within normal limits. Renal function was impaired (urea, 73 mg/dL; creatinine, 1.6 mg/dL), and hypoalbuminemia (2.3 g/dL). Chest radiography showed bilateral pleural effusion, as evidenced by the blunted costodiaphragmatic angles. Electrocardiography demonstrated T-wave inversions in leads V1-V6 and left axis deviation. Echocardiography revealed global left ventricular hypokinesia, severe left ventricular dysfunction, mild tricuspid regurgitation, moderate pulmonary artery hypertension (41 mmHg), and an ejection fraction of 33%. The summary of patient comorbidities and risk profile is mentioned in Table 1.

**Table 1:** Summary of the patient's comorbidities and risk profile CAD: coronary artery disease; CKD: chronic kidney disease; HTN: hypertension; TB: tuberculosis; INR: International normalized ratio; LV: left ventricle; EF: ejection fraction; ASA: American Society of Anesthesiologists

Parameter	Details
Age/Sex	57 years/Female
Primary condition	Infected diabetic foot with osteomyelitis
Urgency of surgery	Emergency (sepsis, non-healing ulcer)
Comorbidities	Diabetes mellitus (30 yrs), CAD, CKD, HTN, spinal TB
Anticoagulation status	On aspirin + clopidogrel
INR	1.6 (elevated)
Hemoglobin	9 g/dL (anemia)
LV function (EF)	33% (severe LV dysfunction)
ASA classification	IVe
Anesthetic contraindications	General anesthesia, neuraxial blockade

Given the patient’s elevated INR, antiplatelet therapy, comorbidities, central neuraxial blockade, and general anesthesia were contraindicated. Ultrasound-guided popliteal sciatic and femoral nerve blocks (PNBs) were planned. The patient was classified as having American Society of Anesthesiologists (ASA) physical status IVe, and informed consent was obtained, acknowledging the risk of intraoperative major adverse cardiac events.

Standard ASA monitors (electrocardiogram, pulse oximetry, non-invasive blood pressure) were used, and an arterial line was secured in the right radial artery for invasive blood pressure monitoring. Supplemental oxygen (5 L/min) was administered, and minimal sedation was achieved with midazolam 0.5-1 mg and fentanyl (50 µg) intravenously.

The patient was positioned prone for a popliteal sciatic nerve block. A high-frequency linear ultrasound probe was placed at the popliteal fossa to identify the popliteal artery and to trace the bifurcation of the sciatic nerve. A 22-gauge Quincke spinal needle was inserted using the in-plane technique, and 20 mL of 0.25% bupivacaine was injected after negative aspiration. For the femoral nerve block, the patient was repositioned supine, and the probe was placed below the inguinal ligament to visualize the femoral artery and vein. Using the same needle and technique, 15 mL of 0.25% bupivacaine was administered after negative aspiration (Table 2).

**Table 2:** Ultrasound-guided peripheral nerve block protocol

Block type	Position	Needle approach	Volume injected	Local anesthetic
Popliteal sciatic block	Prone	In-plane, lateral to medial	20 mL	0.25% bupivacaine
Femoral nerve block	Supine	In-plane, lateral to medial	15 mL	0.25% bupivacaine

Sensory and motor blockade were assessed every 5 min until adequate anesthesia was confirmed. BKA was performed without intraoperative pain or hemodynamic instability. Postoperatively, the patient was transferred to the intensive care unit for further monitoring. Pain was evaluated using the Numeric Pain Intensity Scale (NPIS), with scores of 0 at four hours and three at 24 hours. Sensory and motor functions were normalized within 24 hours (Table 3).

**Table 3:** Intraoperative and postoperative observations NPIS: Numeric Pain Intensity Scale

Parameter	Observation
Intraoperative pain	None
Hemodynamic instability	Absent
Sedation	Midazolam 0.5–1 mg, fentanyl 50 µg
NPIS score (four hours postoperatively)	0
NPIS Score (24 hours postoperatively)	3
Return of sensory/motor function	Within 24 hours
Complications	None

In a high-risk coagulopathic patient undergoing BKA, anesthesia choice must balance safety and efficacy. General anesthesia poses significant cardiovascular risk due to severe left ventricular dysfunction and sepsis. Central neuraxial blockade is contraindicated owing to elevated INR, antiplatelet use, and possible technical difficulty from spinal tuberculosis (TB). While landmark-based PNBs are simple, they carry a higher bleeding risk without visual guidance. Ultrasound-guided PNBs, though technically demanding, allow real-time visualization of nerves and vessels, minimizing vascular injury and maintaining hemodynamic stability. Given the patient’s risks, ultrasound-guided PNB was the safest and most suitable anesthetic approach (Appendix A).

**Figure 1:**
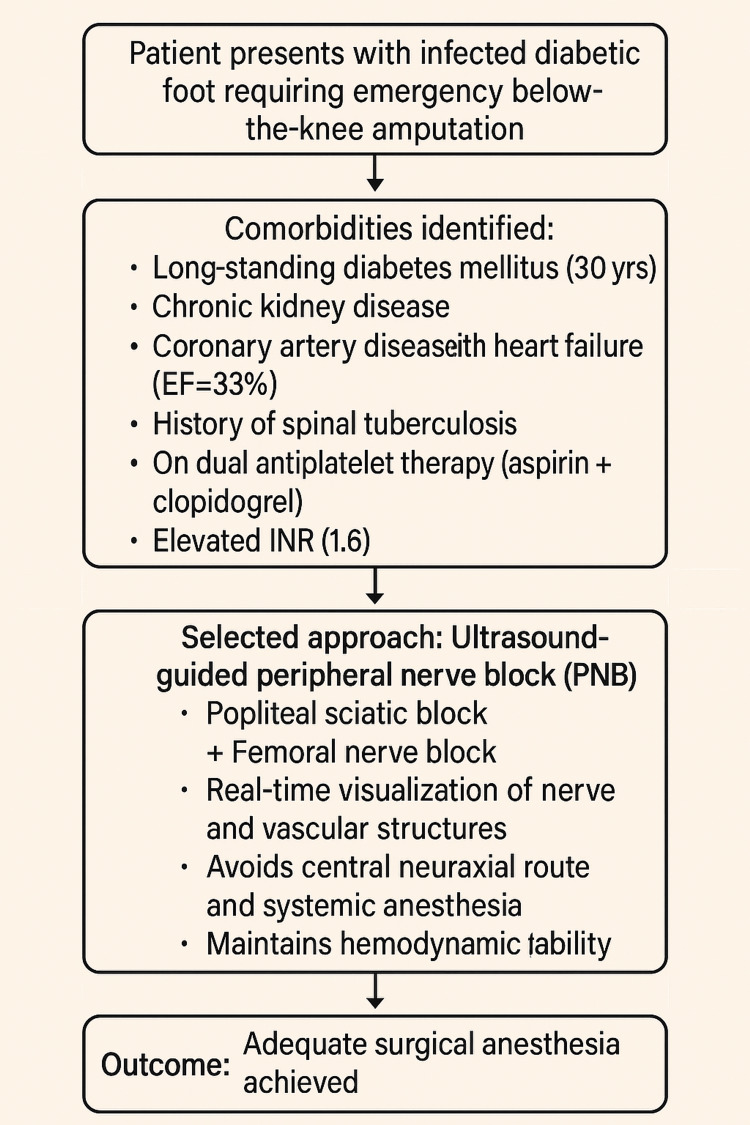
Summary of the workflow EF: ejection fraction; INR: international normalized ratio; +: combination

The workflow diagram (Figure 1) outlines the decision-making process for anesthetic management in a high-risk diabetic patient requiring emergency BKA. The patient had multiple comorbidities, including heart failure, chronic kidney disease, and spinal TB, and was on antiplatelet therapy with elevated INR, ruling out general and neuraxial anesthesia due to high risks. A landmark-based PNB was also unsuitable due to bleeding risks. Ultimately, an ultrasound-guided PNB was chosen for its safety, real-time visualization, and hemodynamic stability. This approach provided effective surgical anesthesia and analgesia without complications, demonstrating an individualized, risk-based anesthetic strategy in complex clinical settings.

## Discussion

Emergency BKA in high-risk patients with coagulopathy and multiple comorbidities requires meticulous anesthetic planning to optimize the perioperative outcomes. In this case, the patient’s elevated INR (1.6), antiplatelet therapy, severe cardiac dysfunction, and history of spinal tuberculosis contraindicated central neuraxial blockade and general anesthesia. Ultrasound-guided PNBs provide a safe and effective alternative, aligning with evidence from clinical studies supporting their use in complex scenarios [4,5,6].

Central neuraxial blockade is contraindicated in patients with an INR > 1.5, as outlined by the American Society of Regional Anesthesia and Pain Medicine (ASRA) guidelines, due to the risk of spinal or epidural hematoma, which can result in permanent neurological deficits [7]. General anesthesia was deemed high-risk in this patient, given her severe left ventricular dysfunction and hypotension. Induction agents and mechanical ventilation may reduce systemic vascular resistance and myocardial contractility, potentially precipitating cardiovascular collapse [8,9]. Additionally, the patient’s history of spinal tuberculosis likely caused vertebral deformities, complicating neuraxial needle placement and increasing the risk of technical failure [10].

Ultrasound-guided PNBs offer significant advantages in these cases. High-frequency ultrasound enables real-time visualization of neural and vascular structures, ensuring precise local anesthetic deposition and minimizing complications such as intravascular injection or nerve injury [4]. A systematic review by Joubert et al., conducted in Canada, analyzed observational studies and case reports and reported a bleeding complication rate of only 0.82% in anticoagulated patients undergoing PNBs, underscoring their safety in surgical settings [11]. Similarly, Ferraro et al., in a Brazilian case series involving orthopedic surgeries, demonstrated that ultrasound-guided PNBs are effective in anticoagulated patients, with negligible bleeding risk due to the direct visualization of vascular structures [5]. In contrast, landmark-based techniques, as studied by Maier et al. in a German cohort of patients undergoing lumbar sympathetic blocks, were less precise and associated with higher complication rates, including severe bleeding in anticoagulated patients [12].

Popliteal sciatic and femoral nerve blocks provided comprehensive anesthesia for BKA, covering the sciatic and femoral nerve distributions. Bupivacaine (0.25%) was selected for its prolonged duration of action, ensuring sustained intraoperative and postoperative analgesia, as supported by a systematic review by Gadsden et al., conducted in the United States, which evaluated local anesthetic concentrations in peripheral nerve blocks [13]. The absence of intraoperative hemodynamic changes and effective pain control (NPIS scores of 0 at four hours and three at 24 hours) highlights the efficacy of this approach. Furthermore, ultrasound guidance mitigated bleeding risks, despite the patient’s elevated INR and antiplatelet therapy, consistent with findings from a large cohort study by Chelly et al. conducted in the United States, which reported favorable outcomes with ultrasound-guided PNBs in high-risk surgical patients undergoing orthopedic procedures [14]. A retrospective study by Patel et al., performed in the United Kingdom, further supports the safety of PNBs in anticoagulated patients, noting minimal hemorrhagic complications in emergency surgeries [15].

This case has limitations, including its single-patient nature, which limits generalizability, and the lack of long-term follow-up data on functional recovery or infection resolution. However, successful intraoperative and immediate postoperative outcomes align with emerging evidence supporting ultrasound-guided PNBs in anticoagulated patients [16]. Future research should focus on prospective multicenter studies to establish standardized protocols for PNBs in high-risk populations, addressing optimal local anesthetic dosing, block techniques, and long-term safety.

## Conclusions

Ultrasound-guided PNB represents a safe and effective anesthetic strategy for emergency BKA in high-risk patients with coagulopathy and multiple comorbidities. By facilitating the precise administration of local anesthetics and minimizing bleeding risks, this approach offers a superior alternative to central neuraxial blockade and general anesthesia, reducing perioperative morbidity and mortality. This case highlights the transformative potential of ultrasound-guided PNBs in complex surgical scenarios and advocates for their broader adoption in clinical practice. Further prospective studies are warranted to develop evidence-based guidelines for its use in patients receiving anticoagulation therapy.

## Appendices

Appendix A

**Table 4:** Comparative analysis of anesthetic options in high-risk coagulopathic patient undergoing BKA LV: left ventricle; EF: ejection fraction; INR: international normalized ratio; TB: tuberculosis, PNB: peripheral nerve block

Anesthetic technique	Advantages	Contraindications/Risks in this case	Suitability
General anesthesia	Rapid induction, familiar technique	Severe LV dysfunction (EF 33%), risk of cardiovascular collapse, septic status	Not suitable
Central neuraxial blockade	Dense anesthesia, effective for lower limb procedures	INR 1.6 (↑ bleeding risk), antiplatelet use, spinal TB (technical difficulty)	Contraindicated
Landmark-based PNB	Can be used without ultrasound, effective if the anatomy is normal	Poor precision, higher risk of vascular injury in anticoagulated patients	High-risk
Ultrasound-guided PNB	Real-time visualization of nerve/vessels, lower bleeding risk, stable hemodynamics	Technically skilled procedure required, time-consuming in emergencies (minor)	Preferred method
